# 86. Effect of Oral Fosfomycin Compared to the Intravenous Beta Lactam/Beta-Lactamase Inhibitor or Carbapenem in Step-down Treatment of Patients with Complicated Urinary Tract Infection due to Extended-Spectrum Beta-Lactamase (ESBL) Producing *Enterobacteriaceae*: A Multicenter, Open-label, Randomized Controlled, Non-inferiority Trial

**DOI:** 10.1093/ofid/ofad500.002

**Published:** 2023-11-27

**Authors:** Jun-won Seo, Da Young Kim, Youngmin Yoon, Young Sang Lyu, Yong Sub Na, Do Sik Moon, Na Ra Yun, Min Seok Kim, Jeong-Han Kim, Sang Woon Bae, Jian Hur, Min Hyung Kim, Yoon Soo Park, Heejung Kim, Hyungdon Lee, Hee-Chang Jang, Dong-Min Kim

**Affiliations:** Chosun university Hospital, Gwangju, Kwangju-jikhalsi, Republic of Korea; Chosun university Hospital, Gwangju, Kwangju-jikhalsi, Republic of Korea; Department of Internal Medicine, College of Medicine, Chosun University, Gwanju, Kwangju-jikhalsi, Republic of Korea; Department of Internal Medicine, College of Medicine, Chosun University, Gwanju, Kwangju-jikhalsi, Republic of Korea; Department of Internal Medicine, College of Medicine, Chosun University, Gwanju, Kwangju-jikhalsi, Republic of Korea; Department of Internal Medicine, College of Medicine, Chosun University, Gwanju, Kwangju-jikhalsi, Republic of Korea; Chosun university hospital, Gwanjugwangyeoksi, Kwangju-jikhalsi, Republic of Korea; Department of Urology, Chosun university college of Medicine, Gwangju, Kwangju-jikhalsi, Republic of Korea; Division of Infectious Diseases, Department of Internal Medicine, Ewha Womans University, Mokdong Hospital, Seoul, Seoul-t'ukpyolsi, Republic of Korea; Division of Infectious Diseases, Department of Internal Medicine, Yeungnam University Medical Center, Daegu, Kyongsang-bukto, Republic of Korea; Yeungnam university hospital, Daejongwanyeoksi, Taegu-jikhalsi, Republic of Korea; Division of Infectious Diseases, Department of Internal Medicine, Yongin Severance Hospital, Yonsei University College of Medicine, Seoul, Seoul-t'ukpyolsi, Republic of Korea; Division of Infectious Diseases, Department of Internal Medicine, Yongin Severance Hospital, Yonsei University College of Medicine, Seoul, Seoul-t'ukpyolsi, Republic of Korea; Department of Laboratory Medicine, Yongin Severance Hospital, Yonsei University College of Medicine, Yongin, Kyonggi-do, Republic of Korea; Chuncheon Sacred heat hospital hallym University, Chuncheon, Kangwon-do, Republic of Korea; Chonnam National University Hospital, Kangju, Kwangju-jikhalsi, Republic of Korea; Chosun University Hospital, Gwang ju, Kwangju-jikhalsi, Republic of Korea

## Abstract

**Background:**

The widespread use of carbapenem in the treatment of UTIs caused by ESBL-producing enterobacteriaceae has led to the emergence of carbapenem resistant enterobacteriaceae. In this randomized controlled trial, we aimed to determine whether oral fosfomycin was inferior to carbapenems or beta-lactamase inhibitors (BL/BLIs) in treatment of complicated UTIs caused by ESBL-producing enterobacteriaceae.

**Methods:**

This study was a multicenter, randomized, controlled, open-label, non-inferiority trial conducted at four tertiary hospitals in South Korea. Eligible patients were randomly assigned 1:1 at the time of step-down therapy, to continue carbapenem or BL/BLIs already in use or to switch to oral fosfomycin (Figure 1). The antibiotic treatment period was set to 10 days in total. The primary end point was clinical resolution of UTIs related symptom and signs within 4 days after the end of treatment. This trial is registered with cris.nih.go.kr (KCT0007669).

**Figure:**
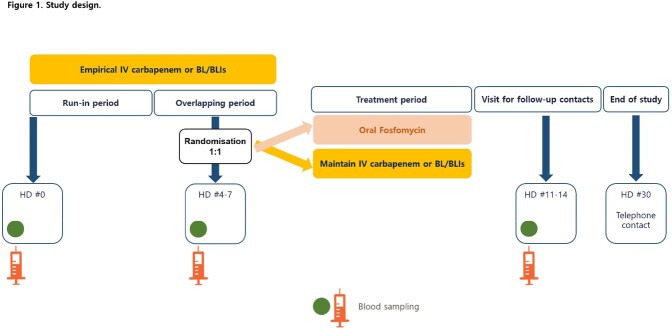

**Figure:**
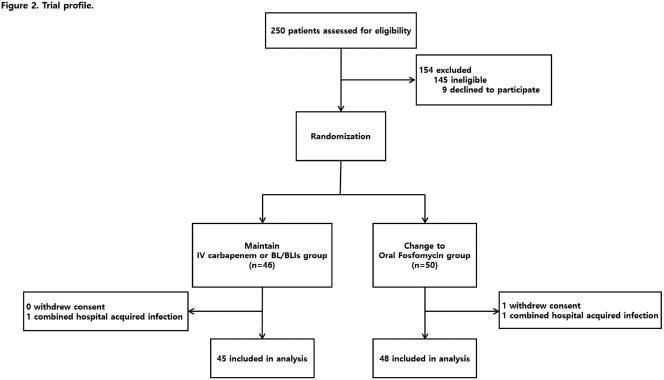

**Results:**

Between Nov 1, 2022, and Apr 30, 2023, 250 patients were screened for eligibility. 96 patients were randomly allocated to receive oral fosfomycin or maintain empirical carbapenem or BL/BLIs, of whom 93 were analyzed (Figure 2). Most of demographic and baseline characteristics showed no statistically significant difference between the two groups (Table 1). The primary endpoint was achieved by 45 (93.8%) of 48 patients in the oral fosfomycin group and 43 (95.6%) of 45 patients in the carbapenem or BL/BLIs group (Figure 3). As a result of the subgroup analysis, there was no statistically significant difference between the two groups for all subgroup items (Figure 4). Microbiological cure in urine specimen was met in 47 of 48 (97.9%) assigned to oral fosfomycin and 44 of 45 (97.8%) assigned to carbapenem or BL/BLIs (Table 2). Side effects were observed in both groups during the treatment period, but the serious adverse events were not observed in both groups (Table 3).

**Figure:**
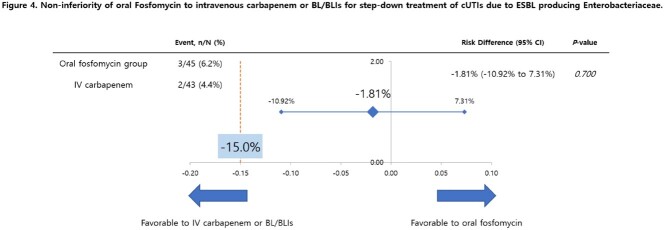

**Figure:**
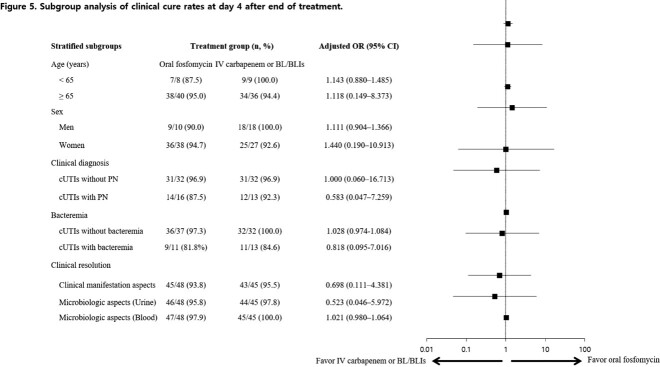

**Figure:**
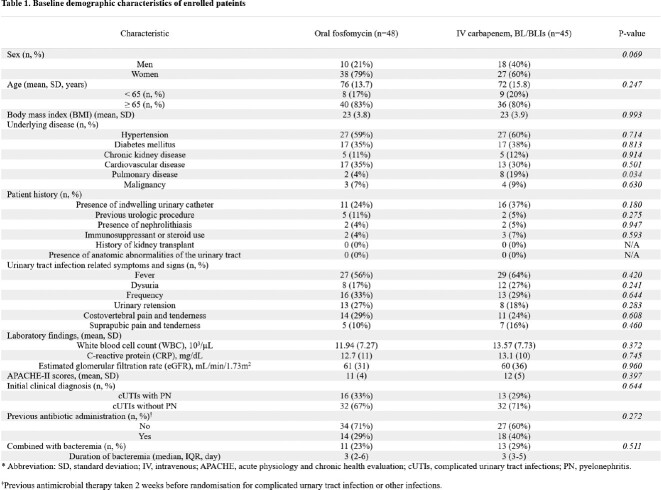

**Conclusion:**

Oral fosfomycin is not inferior to carbapenem or BL/BLIs for the step-down treatment of complicated UTIs caused by ESBL-producing enterobacteriaceae. Therefore, it is one of the optimal treatment options applicable to the strategy of changing to early oral antibiotics in the step-down treatment of complicated UTIs caused by ESBL-producing enterobacteriaceae.

**Figure:**
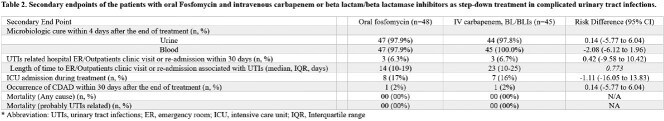

**Figure:**
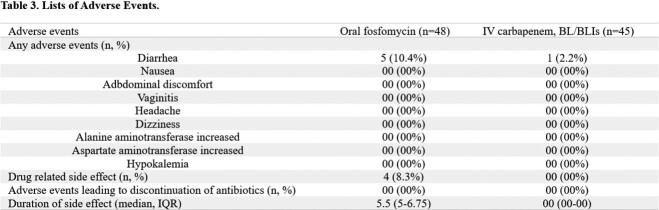

**Disclosures:**

**All Authors**: No reported disclosures

